# Solid Lipid Nanoparticles Hydroquinone-Based for the Treatment of Melanoma: Efficacy and Safety Studies

**DOI:** 10.3390/pharmaceutics15051375

**Published:** 2023-04-29

**Authors:** Sonia Trombino, Rocco Malivindi, Giuseppe Barbarossa, Roberta Sole, Federica Curcio, Roberta Cassano

**Affiliations:** 1 Department of Pharmacy, Health and Nutritional Science, University of Calabria, Arcavacata, 87036 Rende, Italy; sonia.trombino@unical.it (S.T.); rocco.malivindi@unical.it (R.M.);; 2 Azienda Sanitaria Provinciale, Via Alimena, 87100 Cosenza, Italy

**Keywords:** melanoma, solid lipid nanoparticles, hydroquinone, stearic acid

## Abstract

Classical melanoma therapy has several side effects that are responsible for a decrease in the final therapeutic efficacy. It is possible that the drug is degraded before reaching the target site and is metabolized by the body itself, resulting in repeated doses being administered throughout the day and a decrease in patient compliance. Drug delivery systems avoid degradation of the active ingredient, improve release kinetics, prevent the drug from being metabolized before reaching the site of action, and improve the safety and efficacy profiles of adjuvant cancer therapy. The solid lipid nanoparticles (SLNs) based on hydroquinone esterified with stearic acid realized in this work represent a chemotherapeutic drug delivery system that is useful in the treatment of melanoma. The starting materials were characterized by FT-IR and ^1^H-NMR, while the SLNs were characterized by dynamic light scattering. In efficacy studies, their ability to influence anchorage-dependent cell proliferation was tested on COLO-38 human melanoma cells. Furthermore, the expression levels of proteins belonging to apoptotic mechanisms were determined by analyzing the role of SLNs in modulating the expression of p53 and p21WAF1/Cip1. Safety tests were conducted to determine not only the pro-sensitizing potential but also the cytotoxicity of SLNs, and studies were conducted to assess the antioxidant and anti-inflammatory activity of these drug delivery.

## 1. Introduction

Melanoma is a type of malignant tumor that originates from melanocytes in the basal layer of the epidermis and accounts for 1.7% of global cancer diagnoses [1]. The incidence of this cancer has increased, especially in more developed countries, with a predominance in the fair-skinned population [1], and its survival rate is relatively low [2]. Melanoma can be divided into several sub-types, which differ in appearance, incidence and molecular profile [3]. Ultraviolet radiation (UVR) is the main, but not the only, risk factor for the development of cutaneous malignant melanoma (CMM) [4]. In fact, other risk factors related to its occurrence are immunosuppression [5], presence of nevi, genetic predisposition (NRAS gene mutations) [6], and obesity [7]. The treatment of melanoma consists of excision surgery [8], adjuvant therapy with chemotherapy [9], immunotherapy [10], radiation therapy [11], and targeted therapy [12]. Precisely in the context of targeted therapy, an important role is played by solid lipid nanoparticles that would allow delivery of sensitive drugs directly into target lesions in a sufficient concentration to reduce the accumulation of active ingredients in unwanted organs or tissues [13,14].

Active ingredients can be loaded onto the surface of these nanocarriers by a hydrophobic effect, electrostatic interactions, or chemical bonding in order to achieve targeted delivery and enhanced efficacy [15,16,17]. The aim of the present work was to make hydroquinone-based SLNs esterified with stearic acid as carriers for the delivery of biologically active molecules in the treatment of melanoma. The choice of hydroquinone is based on literature data on the ability of this substance to treat skin hyperpigmentation disorders, such as melasma, chloasma, age spots, and sun lentigines [18] and to destroy or inhibit melanoma cells [19]. The presence of stearic acid (SA) or octadecanoic acid is necessary to create a lipid matrix for nanoparticles that is biocompatible with human tissue and neutral to physiological fluids [20,21]. In this work, SLNs were realized using microemulsion techniques and characterized by Dynamic light scattering. The efficacy studies using COLO-38 human melanoma cells, safety tests, and antioxidant and anti-inflammatory activity of these carriers were conducted.

## 2. Materials and Methods

### 2.1. Materials

The following substances were used for the three synthesis reactions: hydroquinone 99%, stearic acid ≥ 98.5% (SA), dichyclohexylcarbodiimide ≥ 99.0% (DCC), 4-dimethylaminopyridine (DMAP), 3,4-dihydro-2H-pyran 97%, distilled water, sodium taurocholate hydrate ≥ 97% (bile salt), butane-1-ol, tween-20, butyroxytoluene (BHT), hydrochloric acid (HCL), tert-butyl alcohol (TBA) and trichloroacetic acid (TCA) purchased from Sigma Aldrich (Saint Louis, MO, USA). The solvents that were used were tetrahydrofuran ≥ 99.0% (THF), chloroform, methanol ≥ 99.8% (CH_3_OH), ethanol (CH_3_CH_2_OH) ≥ 99.8%, and ammonia (NH3) purchased from Sigma Aldrich, VWR Chemical Prolabo and Alfa Aesar. The following materials were used for in vitro safety and efficacy studies on COLO-38 human melanoma cell lines: RPMI 1640 (Life Technologies, Inchinnan, UK), Trypsin (Sigma Aldrich, Milan, Italy), Phosphate-buffered saline (PBS) (Life Technologies, Monza, Italy) Penicillin/Streptomycin (Sigma Aldrich, Milan, Italy), Acrylamide and Bis-Acrylamide (Sigma Aldrich, Milan, Italy), Anti-β-actin antibody (Santa Cruz Biothecnology, Dallas, TX, USA), Anti-PARP1 antibody (Santa Cruz Biothecnology, Dallas, TX, USA), Anti-CD1 antibody (Santa Cruz Biothecnology, Dallas, TX, USA), Anti-Caspase1 antibody (Santa Cruz Biothecnology, Dallas, TX, USA), Phenylmethylsulfonylfluoridre (PMSF) (Sigma Aldrich, Milan, Italy), Bovine serum albumin (BSA) (Sigma Aldrich, Milan, Italy), Bradford reagent (Bio-rad, Hercules, CA, USA), Laemmli buffer, Coomassie brilliant blue (Sigma Aldrich, Milan, Italy), ECL system (Bio-rad, Hercules, CA, USA), Fetal bovine serum (FBS) (Life Technologies, Monza, Italy), L-glutamine (Life Technologies, Monza, Italy), Milk powder (Euroclone), Protease Inhibitor Cocktail Tablets (Roche, Mannheim, Germany), RIPA Buffer (Sigma Aldrich, Milan, Italy), DMEM High Glucose (Sigma Aldrich, St. Louis, MO, USA), HEPES (Sigma-Aldrich, St. Louis, MO, USA), BCS (Sigma Aldrich, St. Louis, MO, USA), Sodium Pyruvate (Gibco, UK), β-mercaptoethanol (Sigma Aldrich, Milan, Italy), MTT (Sigma-Aldrich, St. Louis, MO, USA), DMSO (Sigma Aldrich, St. Louis, MO, USA), DMSO (Sigma Aldrich, St. Louis, MO, USA), TBST 1X, Stacking Solution (pH 6.8), Resolving Solution (pH 8.8), Glycine Buffer, Transblot Buffer (Biorad), COLO 38, BALB/3T3, THP-1, Boyden Chambers, Solid Lipid Nanoparticles (SLN), Vitamin E Ph. Eur. (A.C.E.F., Fiorenzuola D’arda PC, Italy), DAPI (Abcam, Waltham, UK), SDS (Sigma Aldrich, St. Louis, MO, USA), Neutral Red (Sigma Aldrich, Milano, Italy), CD54 (Invitrogen, Waltham, MA, USA), CD86 (Life Technologies, Inchinnan, UK), NiSO_4_ (Sigma Aldrich, Milano, Italy), FACS Buffer (Invitrogen, Waltham, MA, USA), and Propidium Iodide (PI) (Sigma Aldrich, St. Louis, MO, USA).

### 2.2. Instrumentation

Thin-layer chromatography (TLC) was performed using 60 F254 silica gel plates (Sigma Aldrich, St. Louis, MO, USA) on aluminum supplied by Merck using UV light at a wavelength of 254 nm. ^1^H-NMR spectra were carried out using a Bruker VM 30 spectrophotometer (Bruker, Ettlingen, Germany), while FT-IR spectra were recorded using a Jasco 4200 spectrophotometer (Jasco Europe S.R.L, Lecco, Italy). UV-Vis spectra were recorded using a Jasco V-530 UV/Vis (Thermo Fisher Scientific, Monza, Italy) spectrophotometer using 1 cm thick quartz cells. Size analyses of the nanoparticles were carried out using a Brookhaven 90 Plus Particle Size Analyzer with light scattering (Holtsville, NY, USA). Solvent removal was carried out by means of Rotavapor R II Buchi, while freeze-drying of some compounds was performed by means of a Micro Modulyo, Edwards “Freezing-drying”. A ZEISS EM 900 electron microscope (Oberkochen, Germany) at an accelerating voltage of 80 kVwas used to observe nanoparticle morphology.

### 2.3. Hydroquinone Protection Reaction

In a three-necked flask fitted with a reflux condenser, carefully flamed and kept under nitrogen, 15 mL ethyl ether, 0.043 g para-toluenesulphonic acid and 0.5 g hydroquinone were placed. Immediately after dissolution of the hydroquinone, 0.35 mL of 3,4-dihydro-2H-pyran was added by means of a dropping funnel into 5 mL of diethyl ether and the mixture was stirred for 48 h. At the end of the reaction, the mixture was washed with an aqueous ammonia solution and the organic phase, extracted by means of dichloromethane, was purified by Merk 60 silica gel column chromatography (70–230 mesh) using n-hexane/ethyl acetate 7/3 as an eluent. The purified product was characterized by NMR (Nuclear Magnetic Resonance) spectroscopy and IR (Infrared Radiation) spectroscopy.

### 2.4. Synthesis of Hydroquinone Monostearate

Stearic acid, THF and hydroquinone monostearate were placed in a three-necked flask equipped with a reflux condenser, carefully flamed and kept under nitrogen. Immediately after dissolution of the hydroquinone, DCC and DMAP were added in sequence and stirred for 24 h. The reaction was filtered using a porosity 4 filter and then taken to the rotavapor for the removal of the remaining solvent and the by-product dicyclohexylurea [22,23]. The reaction was conducted in accordance with the procedure reported in the literature. Finally, the reaction product was thoroughly purified by Merk 60 silica gel flash column chromatography (70–230 mesh) using CH_2_Cl_2_/*n*-hexane 9/1 as the eluent. The purified product (0.92 g) was characterized by Nuclear Magnetic Resonance (NMR) and Infrared Radiation (IR) spectroscopy.

### 2.5. Deprotection of Hydroquinone Monostearate

Hydroquinone monostearate was added with 20 mL of ethanol, acidified with a concentrated HCl solution and extracted with 150 mL of dichloromethane in order to obtain the deprotected hydroquinone monostearate useful for making solid lipid nanoparticles. The combined organic phases were anhydrified and subjected to purification using a chromatographic column (Merk silica gel 60–70/230 mesh, n-hexane/ethyl acetate 8/2). The product obtained was characterized using common spectroscopic techniques.

### 2.6. Preparation of SLNs by the Microemulsification Method

The fabrication of solid lipid nanoparticles can be carried out by microemulsification, which consists of the initial mixing of the hot ester with the surfactants (Tween 20, butanol and sodium taurocholate) at 70 °C. The nanoparticles are formed by dispersing the resulting mixture in cold water under mechanical agitation at high speed [24]. The substances used are reported in Table 1.

### 2.7. Characterization of SLN

An aliquot of nanoparticles (10 µL) dispersed in distilled water was characterized by Dynamic Light Scattering to determine their average diameter, polydispersion index and potential zeta. Particle size measurements were carried out at 25 °C. The morphology of these was assessed using TEM. A small amount of solution was applied to a carbon-coated copper grid and left for a few minutes to promote the adhesion of the nanoparticles to the carbon substrate. A drop of 1% phosphotungstic acid solution was applied and the air-dried sample was observed with a ZEISS EM 900 electron microscope at an accelerating voltage of 80 k. Stability studies were conducted at room temperature (25 °C) and refrigerated (4 °C) for 60 days.

### 2.8. Cell Lines and Culture Conditions

The COLO-38, Balb/3T3 Clone A31 and THP-1 cell lines were purchased from ATCC, Manassas, VA, USA. COLO-38 cells, derived from amelanotic melanoma, were cultured in RPMI 1640 1× medium supplemented with 10% Fetal Bovine Serum (FBS), 1% Penicillin/Streptomycin, 1% L-Glutamine and 25 mM HEPES (4-2-hydroxyethyl-1-piperazinyl-ethanesulphonic acid) buffer. Balb/c 3T3 clone A31 cells, murine fibroblasts, were maintained in DMEM with 10% Calf Bovine Serum (CBS; ATCC, Manassas, VA, USA) and 1% penicillin-streptomycin (10,000 unit/mL). THP-1 cells, derived from a monocyte cell line isolated from the peripheral blood of a patient with acute leukemia, were cultured in RPMI 1640 medium with 10% Fetal Bovine Serum (FBS), 1% Penicillin/Streptomycin and 0.05% mM β-Mercaptoethanol. All cell lines were maintained at 37 °C in modified air containing with 5% humidified CO_2_.

### 2.9. Cell Viability Assay

Cell viability was measured with the 3-(4,5-dime-thylthiazol-2-yl)-2,5-diphenyltetrazolium (MTT) assay. The cells were seeded in 48-well plates at a density of 4 × 10^4^ for COLO-38 melanoma cells and synchronized in serum-free media (SFM) for 12 h. The cells were treated with increasing doses of SLN. After 24 h, 200 µL of MTT (5 mg/mL) was added to the cell media for 2 h at 37 °C. Finally, 200 µL of DMSO was added to each well, and the optical density was measured at 570 nm using a Beckman Coulter microplate reader [25]. Eight replicates were performed for each sample.

### 2.10. Protein Extraction and Western Blot Analysis

The cells were grown in 100 mm dishes to 70–80% confluence and treated with media containing SLN (5 μL/mL). After 24 h of treatment, the cell monolayers were washed with cold PBS and solubilized in a RIPA buffer (Cell Signaling Technology, Danvers, MA, USA) with 1 mM of phenylmethanesulfonyl fluoride (PMSF). The cell lysates were quantified spectrophotometrically using the Bio-Rad Bradford Assay (Bio-Rad Laboratories, Hercules, CA, USA). All of the samples were loaded on a 13% SDS–polyacrylamide gel, transferred to a nitrocellulose membrane, and probed with antibodies directed against PARP-1 (sc-8007), p53 (sc126) and p21^Cip/WAF1^(sc-6246) (Santa Cruz Biotechnology, Santa Cruz, CA, USA). As the internal control, all of the membranes were probed with anti-glyceraldehyde-3-phosphate dehydrogenase, β-Actin (sc47778) antibody (Santa Cruz Biotechnology). The antigen–antibody complex was detected through incubation of the membranes for 1 h at room temperature with peroxidase-coupled goat anti-mouse, anti-rabbit or anti-goat IgG and revealed using the enhanced chemiluminescence system (Santa Cruz Biotechnology). The blots were then exposed to film (Santa Cruz Biotechnology). The intensity of bands representing the relevant proteins was measured using Image J densitometry scanning software [26].

### 2.11. Wound-Healing Scratch Assay

COLO-38 cells were grown to confluence in regular media and then maintained in SFM for 12 h. The monolayers were scratched as previously described [27] and treated with SLN (5 μL/mL). Then, wound healing was photographed at 24 h at 4× magnification using phase-contrast microscopy (CKX-53 Olympus).

### 2.12. Transmigration Assay

COLO-38 cell lines were treated as indicated and placed in the upper compartments of a Boyden chamber (8 mm membranes; Corning Costar, NY, USA), as previously reported. After 12 h, the migrated cells were fixed and stained with 4′,6-diamidino-2-phenylindole (DAPI). Migration was quantified by viewing five separate fields per membrane at 10× magnification (BX-51 Olympus microscope) and were expressed as the mean number of migrated cells.

### 2.13. Neutral Red Uptake Assay

The in vitro NRU test was described by ISO 10993-5:2009 “Biological evaluation of medical devices-Part 5: Tests for in vitro cytotoxicity” on Balb/3T3 Clone A31 cells. Cells with dimensions of 2.5 × 10^4^ 3T3 were treated with increasing doses of SLNin DMEM for 24 h at 37 °C and 5% CO_2_ atmosphere. Cell viability was evaluated by a neutral red uptake (NRU) assay, which included incubation (3 h) with neutral red solution (50 µg/mL), followed by extraction with acetic acid, ethanol and water (1:50:49 *v*/*v*/*v*) [28].

The absorbance was measured at 540 nm using a microplate reader Epoch (BioTek, Winooski, VT, USA). A percentage of viability was calculated as follows:

%Viability = [Abs (540 nm) _test material_ − Abs (540 nm) _blank_]/[Abs (540 nm) _control_ − Abs (540 nm) _blank_]

### 2.14. h-CLAT Activation Test

The purpose of the h-CLAT Assay (Human Cell Line Activation Test) is to clarify whether substances or mixtures cause activation of the immune system, thus resulting in skin sensitization in accordance with the method described by the Organization for Economic Cooperation and Development (OECD) 442E [29] and in the 158 EURL-ECVAM protocol (European Union Reference Laboratory for alternatives to animal testing). The test is performed on THP-1 monocyte cells, on which the modulation of the expression of CD54 and CD86, two costimulatory molecules, is assessed using nickel sulfate (NiSO_4_) as a positive control. The increase in CD54 and CD86 on monocytes correlates with the activation of an immune response following exposure to a partially allergenic antigen. THP-1 cells were cultured in RPMI 1640 medium with 10% Fetal Bovine Serum (FBS), 1% Penicillin/Streptomycin and 0.05% mM β-Mercaptoethanol, plated in a 96-well multiwell at a concentration of 1.5 × 10^5^ cells per well. After 24 h, the cells were centrifuged and the one containing the samples and controls was added. The next day, the cells were centrifuged and re-suspended in FACS buffer in the presence of PI (Propidium Iodide) for analysis in the cytofluorometer. The CV75, i.e., the concentration causing 25% mortality, was then calculated to be used later for the actual test. Subsequently, the sample was solubilized in phosphate buffer at a concentration of 100× the 1.2× CV75. Three stock solutions were therefore prepared, with serial 1:1.2 dilutions, in phosphate buffer, from 0.335× CV75 to 1.2× CV75. These were then diluted 50-fold in the culture medium and tested on the cells directly with a further 1:2 dilution. The positive control was NiSO_4_ at a concentration of 100 μg/mL, while the culture medium was used as the negative control. The experiment was repeated on 3 different days and conducted in 3 replicates. After exposure, the cells were centrifuged and re-suspended in FACS buffer and then divided into three aliquots. They were then centrifuged, re-suspended in blocking solution (FACS Buffer containing 0.01% γ globulin), and subsequently incubated for 15 min at 4 °C. Finally, the cells were labeled with a fluorescein antibody targeting CD86, CD54 or IgG1, with the latter used as a control, for 30 min at 4 °C. Washes with FACS buffer were performed and additional FACS buffer with propidium iodide (PI) was added. Expression of CD54, CD86 and cell viability levels were then assessed in the cytofluorometer, and the results were calculated as previously described [30].

### 2.15. Evaluation of Antioxidant Activity

The antioxidant activity of SLN was evaluated on liver microsomal membranes of murine origin. These have the characteristic of being the ideal substrate of lipid peroxidation processes as they are composed of a large number of polyunsaturated fatty acids, which are degraded in this process. Reactive oxygen species, as a result of degradation, give rise to aldehydes and one of these is malondialdehyde, which is used as a biomarker to measure the level of oxidative stress present in an organism. In order to assess the antioxidant properties, tert-butylhydroperoxide (t-BOOH) has been used as an inducer of oxidative stress [31].

### 2.16. Inhibition of Nitroxide Production on the RAW 264.7 Cell Line

The ability of the samples to inhibit nitroxide production was estimated in vitro using the RAW 264.7 cell line (murine macrophages). The cells were cultured in Dulbecco’s Modified Eagle’s Medium (DMEM), to which 10% fetal serum was added. 10% fetal bovine serum (FBS), 1% L-glutamine and 1% penicillin/streptomycin solution. The cultures were placed at temperature of 37 °C in the presence of 5% CO_2_ in a humidified atmosphere. Cell counts were performed using trypan blue. The cells were then placed in culture in 96-well plates (1 × 10^5^ cells/well), which were used for the experiments twenty-four hours later. The cell cultures were then incubated for 24 h in the presence of different concentrations of the test samples and lipopolysaccharide (LPS, final concentration 1 μg/mL). After 24 h, the supernatant was taken from each well, placed in a new plate and 100 μL of Griess’ reagent (1% sulphamide and 0.1% N-naphthyl) was added ethylenediaminedihydrochloride in 2.5% H_3_PO_4_). Griess’ reagent is a liquid normally almost colorless or off-white, but in the presence of nitrites, it acquires a dish color. Absorbance values were determined at a wavelength of 490 nm [21]. To verify the absence of cytotoxic effects of the extract on the cell line employed, an MTT test was performed [32]. At the end of the incubation period, the medium was removed and 100 μL of MTT 0.5% *w*/*v* was solubilized in DPBS (Dulbecco phosphate baffer saline). After four hours, the MTT solution was removed and 100 μL of DMSO was added to each well. The absorbance was read at 490 nm using a microplate reader. The percentage inhibition of cell viability was assessed according to the following formula:

% inhibition = (1 − sample absorbance/control absorbance) × 100

### 2.17. Statistical Analysis

In vitro data were analyzed by Student’s *t*-test using the GraphPad Prism 8.3.0 (GraphPadSoftware, Inc., San Diego, CA, USA). *p* < 0.05 was considered statistically significant.

## 3. Results

### 3.1. Synthesis and Characterization of Esters

**Scheme 1:**
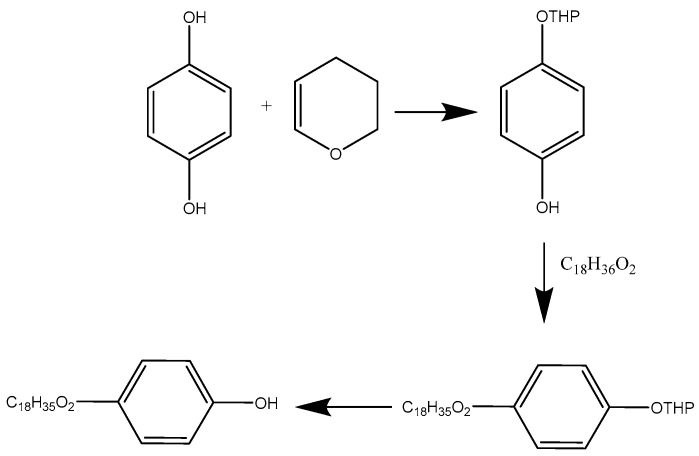
Hydroquinone and stearic acid esterification.

The esterification reaction between hydroquinone and stearic acid was conducted using monoprotected hydroquinone as the starting substrate [yield 51%; FT-IR (KBr) 3346, 3110, 3080, 1509, 1180; 1H-NMR (CD3OD), δ (ppm) 1.59–1.99, (m, 6H), 3.57 (m, 1H), 3.92 (m, 1H), 5.21 (t, 1H), 6.68 (d, 2H), 6. 87 (d, 2H)] in order to obtain a long carbon chain substrate (yield 51%; FT-IR (KBr) 2927, 2852, 1725, 1509; ^1^H-NMR (CDCl3), (ppm) 1H NMR: δ 0.86 (3H, t), 1.16–1.34 (28H, m), 1.56 (2H, tt), 1.99–2.17 (4H, m), 2. 40 (2H, t), 3.50–3.68 (4H, m), 4.42 (1H, tt), 6.89–7.04 (4H, m)] to be used, after deprotection, as a lipid matrix in the preparation of SLNs, potentially useful carriers for the delivery of molecules with anti-tumor activity. The synthesis was conducted in anhydrous tetrahydrofuran (THF) at room temperature, with 70% yield (Scheme 1).

**Figure 1:**
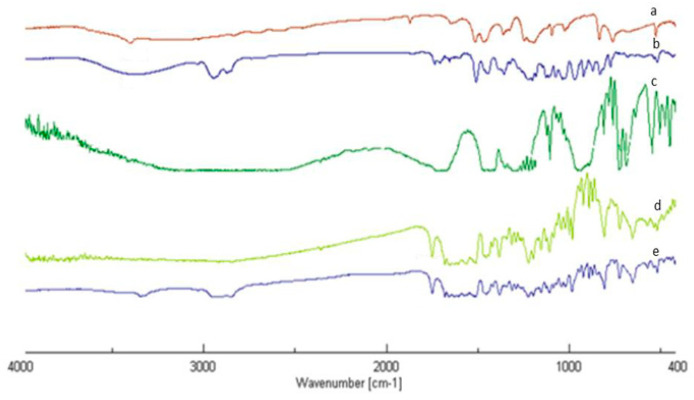
FT-IR Spectrum of: Hydroquinone (**a**), Hydroquinone protected (**b**), Stearic acid (**c**), SA-hydroquinone ester protected (**d**), SA-hydroquinone ester deprotected (**e**).

The formation of deprotected ester was also confirmed by FT-IR and ^1^H NMR (Figure 1). In particular, the FT-IR spectrum of the ester (d), compared with those of the starting substances (a–c) shows the presence of a new band at 1749 cm^−1^ that can be attributed to the stretching vibration of the C=O group of the ester. In this spectrum, we did not observe the bands typical of the stretching vibrations of phenolic OHs visible in the spectrum of the deprotected ester (e) at 3354 cm^−1^. In addition, typical bands of the stretching vibration of aromatic CHs between 3110 and 3025 cm^−1^ were observed.

The ^1^H-NMR spectrum confirmed deprotection by exhibiting both the proton signals of the aromatic rings of the hydroquinone and those of the aliphatic chain of stearic acid: ^1^H NMR ((CD3OD)): δ 0.86 (3H, t), 1.16–1.36 (28H, m), 1.56 (2H, tt), 2.40 (2H, t), 6.87–7.12 (4H, m).

### 3.2. Preparation and Characterization of Solid Lipid Nanoparticles Based on Hydroquinone Monostearate

**Figure 2:**
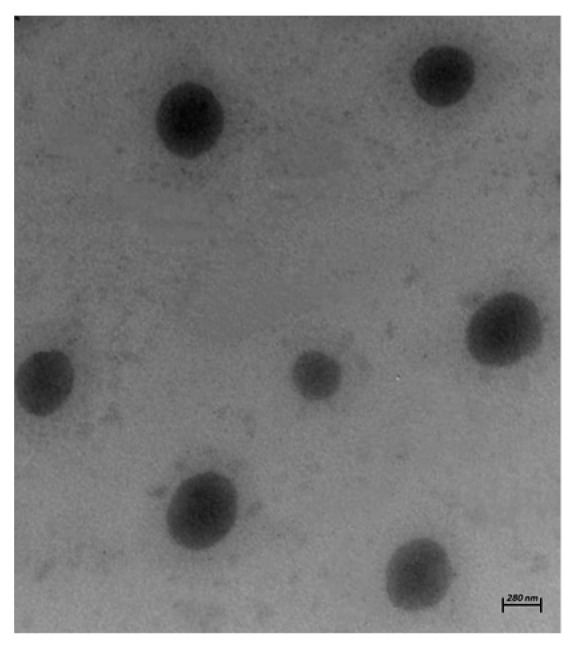
TEM photomicrography of SLN based on hydroquinone monostearate.

SLNs were successfully prepared using the microemulsion technique. Analysis by Dynamic Light Scattering enabled the determination of the average diameter of the nanoparticles and their polydispersion index (PI), as shown in Table 2. These PI values were indicative of good homogeneity in the particle size distribution. The results from TEM characterization showed that SLNs possessed a spherical shape, with dimensions ranging from approximately 250 nm to 300 nm (Figure 2). The obtained results from SLN stability indicated that negligible changes were observed in all measured parameters, maintaining good stability for approximately one month.

### 3.3. Inhibition of Cell Proliferation in SLN-Treated COLO-38 Melanoma Cells

**Figure 3:**
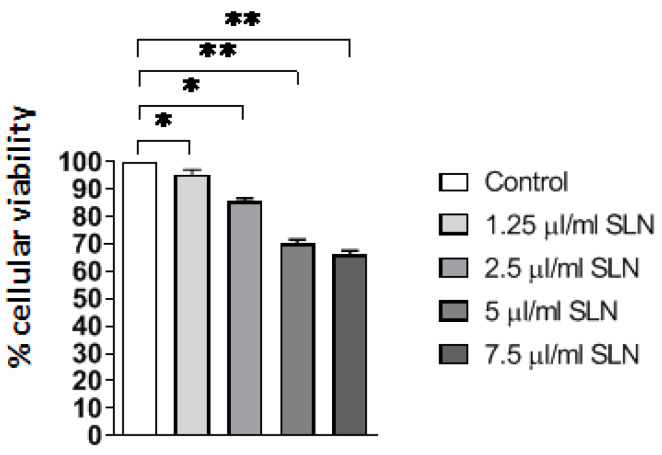
MTT cell proliferation assay in COLO-38 melanoma cancer cells. MTT proliferation assay conducted on COLO-38 cells treated with increasing doses of SLN for 24 h. Results are expressed as a percentage of mean absorbance values, compared to control, and represent the mean ± SE of 3 different experiments. * *p* < 0.01; ** *p* < 0.001 compared to control.

After performing the chemical characterization studies on SLN, we investigated whether these compounds could influence cell proliferation in our experimental model, COLO-38 cells, using the anchorage-dependent assay, MTT. Our results showed that treatment with increasing doses of SLN (1.25, 2.5, 5 and 7.5 μL/mL) decreased the incorporation of the substrate 3-(4,5-dimethylthiazol-2-yl)-2,5-diphenyltetrazolium bromide (MTT). These data demonstrate that SLNs are able to reduce the proliferation of COLO-38 melanoma cancer cells in a dose-dependent manner (Figure 3).

### 3.4. SLN Treatment of COLO-38 Melanoma Cells Causes Death by Apoptosis In Vitro

**Figure 4:**
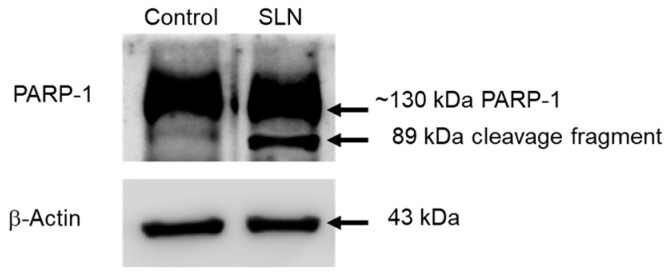
Increased PARP cleavage after SLN treatment. Protein extracts of COLO-38 melanoma cells treated with a dose of SLN (5 μL/mL) for 24 h were subjected to Western blotting analysis in order to assess the expression of PARP-1. β-Actin was used as a loading control.

Confirming what we reported, we investigated whether the decreased cell growth, in our experimental model, induced by the use of hydroquinone-based SLNs, could be caused by mechanisms of programmed death, i.e., apoptosis. For this purpose, we evaluated the expression levels of the main proteins involved in apoptotic mechanisms using Western blot analysis. The COLO-38 melanoma cell line was treated with SLN (5 μL/mL) for 24 h, and our results showed an increase in the cleaved forms, in both treatments, of a well-known substrate of effector caspases, namely ADP RIBOSIUM PO-LYMPHYRASE (PARP) (Figure 4).

### 3.5. Up-Regulation of p53 and p21 Expression in SLN-Treated COLO-38 Melanoma Tumour Cells

**Figure 5:**
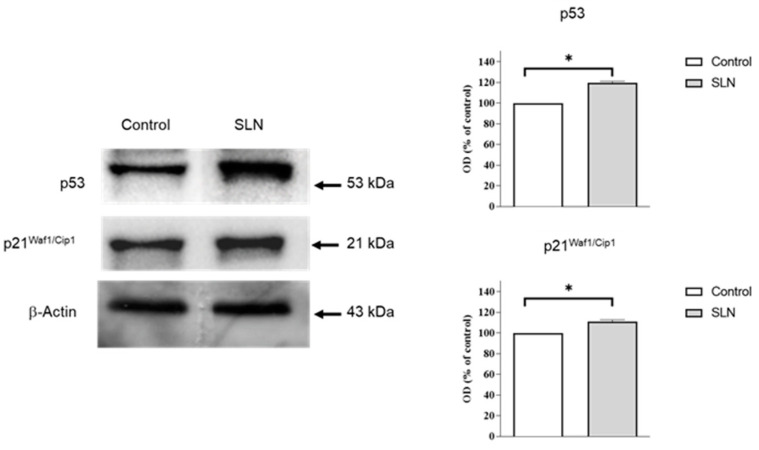
BTA-mediated up-regulation of p53 and p21WAF1/Cip1 expression. Protein extracts of COLO-38 melanoma cells treated with a dose of SLN (5 μL/mL) for 24 h were subjected to Western blotting analysis in order to assess the expression of p53 and p21WAF1/Cip1. β-Actin was used as a loading control. Data are expressed as means ± SD relative to control of 3 separate experiments in which band intensities were evaluated in terms of optical density arbitrary units and expressed as percentages of the control, which was assumed to be 100%. * *p* < 0.01 compared to control.

To better understand the molecular mechanisms responsible for apoptotic events mediated by solid lipid nanoparticles in COLO-38 melanoma cells, we evaluated the ability of SLNs to modulate the expression of p53, an onco-suppressor protein that plays a key role in apoptotic events, as well as the corresponding p21WAF1/Cip1 target gene. The results showed an increase in the protein expression levels of both proteins in our experimental models (Figure 5).

### 3.6. SLNs Induce Increased Motility in COLO-38 Melanoma Cells

**Figure 6:**
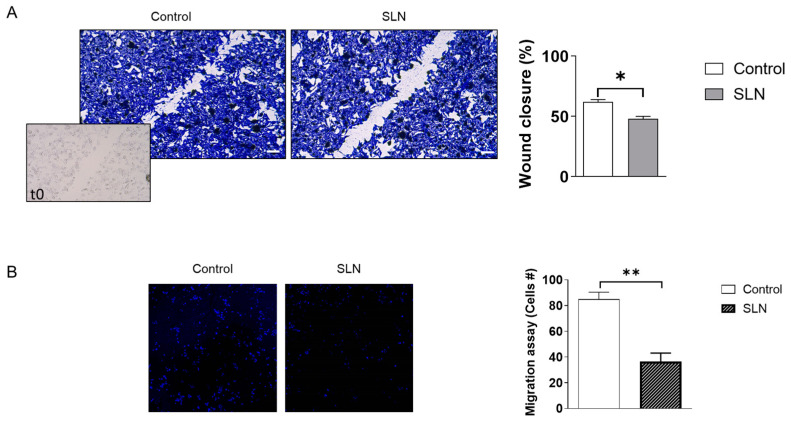
Effects of solid lipid nanoparticles on motility in melanoma cells. (**A**) Wound Healing Scratch Assay in cells treated with SLN as is (5 μL/mL). After 24 h of treatment, the cells were stained with Brilliant Blue Coomassie and then photographed under an OLYMPUSBX 51 microscope at 10× magnification. The histogram represents the relative percentage of cut closure, calculated by image analysis using ImageJ software. Scale bar: 100 μm. (**B**) The Boyden Chamber Transmigration assay was used to assess the transmigratory activity of COLO-38 melanoma cells treated with SLN (5 μL/mL). After 12 h, the migrated cells were fixed and stained with DAPI (4′,6-diamidin-2-phenylindole), with nuclear localization. Migration was assessed by visualizing 6 different fields for each membrane at 10× magnification and expressed as the number of migrated cells in three independent experiments. * *p* < 0.01; ** *p* < 0.001.

Consequently, we investigated what effects SLNs had on cell motility, using the Wound Healing Assays and Transmigration assay. For this reason, COLO-38 melanoma cell lines were starved using serum-free medium for 24 h. A scratch was performed when the confluence was 100%, and they were subsequently treated with SLN (5 μL/mL). Cell motility was observed under a microscope and photographed after 24 h. The COLO-38 cells treated with SLN showed reduced motility in both directions to close the scratch. The results are shown in Figure 6A. This result was confirmed by the Boyden Chambers Migration Assay (Figure 6B). He demonstrated that treatment with SLN for 12 h was able to reduce not only motility but also the migration of the COLO-38 cell lines.

### 3.7. Evaluation of Cytotoxicity: Neutral Red Uptake (NRU)

**Figure 7:**
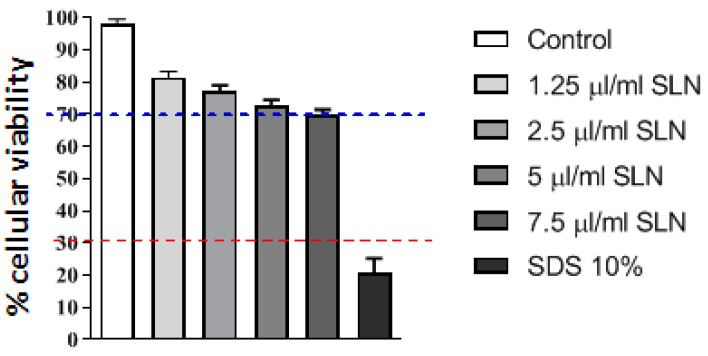
NRU test results. Balb/3T3 Clone A31 fibroblast cell viability (%) measured through NRU cytotoxicity assay upon treatment with increased concentration of SLN. Each column represents the means ± SD of 3 wells/group. RED LINE: strongly cytotoxic, cellular viability < 30%, BLUE LINE: not cytotoxic, 100 < Cellular viability < 70%.

After conducting efficacy studies of the solid lipid nanoparticles, safety studies were conducted to assess any cytotoxic and/or pro-sensitizing effects of our compounds. The effect of SLN on Balb/3T3 Clone A31 fibroblast cells was measured using an in vitro acute toxicity assay through neutral red uptake (NRU, Figure 7) according to ISO 10993. The treatment with increased concentrations of SLN non-altered cell viability compared to the control.

The qualitative assessment was carried out after 24 h of incubation using an inverted microscope to determine the cell integrity. Biological reactivity (including cell degeneration and malformations) was assigned according to the scoring system from 0 to 4 reported in ISO 10993-5, the results are shown in Table 3 and Table 4.

Since reaching a numerical degree above 2 is considered a cytotoxic effect, it can be inferred that this evaluation confirmed the non-toxicity of the compounds examined.

### 3.8. In Vitro Analysis of Pro-Sensitising Potential (h-CLAT)

The aim of the h-CLAT test is to assess whether a product is a skin sensitizer or not. This assay involves the use of a THP-1 cell line to determine the expression level of two co-stimulatory molecules, such as CD54 and CD86. An increased expression level of these molecules on monocytes, indeed, is associated with the activation of the immune response following exposure to potentially sensitizing contact allergens.

First, cell viability was assessed by propidium iodide (PI) uptake, while the concentration showing 75% THP-1 cell survival (CV75) was calculated by log-linear interpolation and used to determine the highest concentration of the tested product to be used in CD86/CD54 expression measurement in the h-CLAT test. Then, THP-1 cells were treated with different serial dilutions of SLN and the relative fluorescence intensity (RFI) of CD86 and CD54, which is proportional to the expression level of the two co-stimulatory molecules, was determined for positive control cells and treated cells.

If the RFI% value of CD86 is equal to or greater than 150% at any tested dose (>50% of cell viability) in at least two independent runs and/or if the RFI of CD54 is equal to or greater than 200% at any tested dose (>50% of cell viability) in at least two independent runs, the sensitization prediction is considered positive. Otherwise, it is considered negative [33]. The obtained results (Table 5) confirmed that the SLN did not show any sensitizing potential.

### 3.9. Evaluation of Antioxidant Activity

**Figure 8:**
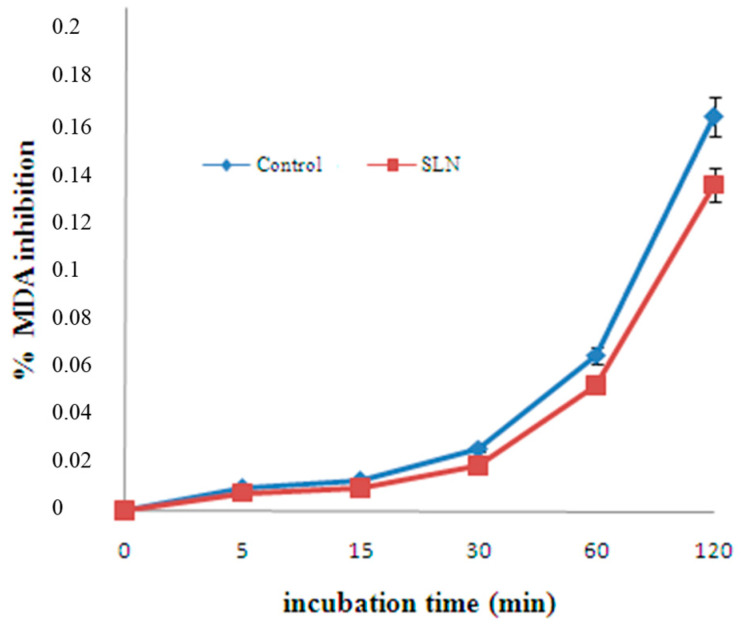
SLN antioxidant activity.

The ability of SLNs to inhibit lipid peroxidation, induced by a free radical generator such as *tert*-BOOH, was examined in rat liver microsomal membranes over a 120-min incubation period. SLNs were able to inhibit lipid peroxidation in a time-dependent manner (Figure 8).

### 3.10. Inhibition of Nitroxide Production on the RAW 264.7 Cell Line

The inhibition of nitroxide production by RAW 264.7 cells was assessed by determining the amount of nitrite, a stable oxidized product of NO, by inducing an inflammatory stimulus using *E*. *coli* lipopolysaccharide (LPS). Preliminary results showed that SLNs have no inhibitory activity against the production of 39 nitroxide production in macrophage cells when stimulated with LPS, as confirmed by literature data.

## 4. Conclusions

The Solid Lipid Nanoparticles (SLNs), which were studied in this research, are a type of nanoparticulate drug delivery system that is useful in the treatment of melanoma. The focus of this study was on the characterization of the safety and efficacy of SLN in human melanoma cells (COLO-38). Specifically, SLNs were synthesized using hydroquinone esterified with stearic acid, with the future goal of delivering chemotherapeutic drugs in oncology therapy.

In terms of efficacy, we tested the ability of SLNs to influence anchorage-dependent cell proliferation. Treatment with increasing doses of SLNs decreased the proliferation of human melanoma tumor cells in a dose-dependent manner.

Based on these scientific findings, we questioned whether apoptosis could be the basis for the blockage of cell growth caused by SLNs in the experimental models. Therefore, the levels of protein expression belonging to apoptotic mechanisms were determined. The observed results demonstrated an increase in the cleaved forms of PARP, whose function is to identify single-stranded DNA lesions.

We also analyzed the role of SLNs in modulating the expression of p53, a protein belonging to tumor suppressors and a key element in apoptotic pathways, as well as p21^WAF1/Cip1^, a target gene of p53. The results allowed us to establish a dose-dependent increase in the expression levels of these proteins in the experimental models examined. The proliferation results highlighted how treatment with SLNs reduced the growth of COLO-38 melanoma cells. We further studied the effects of these nanoparticulate systems on cell motility using the Boyden Chambers Transmigration Assay and Wound Healing Scratch Assay. The results revealed that Solid Lipid Nanoparticles can block the motility of COLO-38 cell lines.

Subsequently, our research focused on safety testing to determine not only the potential for sensitization but also the cytotoxicity of SLNs. Regarding cytotoxicity, we used the Neutral Red Uptake (NRU) test according to ISO 10993-5:2009, “Biological Evaluation of Medical Devices”—Part 5: Test for In Vitro Toxicity, which showed that the sample was not cytotoxic. Finally, we studied the potential for sensitization in vitro using the h-CLAT method (Human Cell Line Activation Test, OECD-442E), which revealed that there was no sensitizing action. SLNs exhibited antioxidant activity in a time-dependent manner but did not have inhibitory activity of nitroxide production on the RAW 264.7 cell line. Therefore, the results obtained confirmed not only the efficacy and safety of Solid Lipid Nanoparticles (SLN) but also how these particles based on hydroquinone esterified with stearic acid could represent an excellent drug delivery system capable of being used in the treatment of melanoma as a carrier of various chemotherapeutics. Further preclinical and clinical studies are needed to determine whether melanoma therapy at the nanoscale could be a breakthrough in the battle against metastatic melanoma.

## Figures and Tables

**Table 1:** Amount of reagent used in SLN preparation.

Ester (g)	Tween 20 (mL)	Butanol (mL)	Sodium Taurocholate (g)
0.05	0.044	0.018	0.023

**Table 2:** SLN dimensional analysis.

Formulation	Size (nm)	Polydispersion Index (PI)
hydroquinone monostearate SLN	289 ± 5.3	0.005

**Table 3:** Results Interpretation.

Grade	Reactivity	Condition of All Cultures
0	None	No detectable areas around or under the sample
1	Slight	Some malformed or degenerated cells under the sample
2	Mild	Limited area under the sample
3	Moderate	Area under the sample extending up to 1.0 cm
4	Severe	Area extending more than 1.0 cm outside the sample

**Table 4:** Assessment of biological reactivity.

Sample	Biological Reactivity
Control	0
0.125 μL/mL SLN	0
0.25 μL/mL SLN	0
0.5 μL/mL SLN	1
0.75 μL/mL SLN	2
Control + (SDS 10%)	4

**Table 5:** Analysis conducted on THP-1 monocyte results.

Samples	CD54 *	CD86 *
0.25 μL/mL SLN	46.37	57.17
0.5 μL/mL SLN	51.23	66.12
0.75 μL/mL SLN	59.62	69.12
Control	38	57
Control + (NISO_4_)	180	221

* The compound is skin sensitizer where CD86 > 150 and CD54 > 200.

## Data Availability

Not applicable.
